# Levamisole suppresses adipogenesis of aplastic anaemia‐derived bone marrow mesenchymal stem cells through ZFP36L1‐PPARGC1B axis

**DOI:** 10.1111/jcmm.13761

**Published:** 2018-07-11

**Authors:** Lu‐Lu Liu, Lei Liu, Hai‐Hui Liu, Sai‐Sai Ren, Cui‐Yun Dou, Pan‐Pan Cheng, Cui‐Ling Wang, Li‐Na Wang, Xiao‐Li Chen, Hao Zhang, Ming‐Tai Chen

**Affiliations:** ^1^ Central Laboratory Affiliated Hospital of Jining Medical University Jining China; ^2^ Department of Hematology Affiliated Hospital of Jining Medical University Jining China; ^3^ Department of Graduate School Jining Medical University Jining China

**Keywords:** adipogenic differentiation, apalastic anaemia, levamisole, mesenchymal stem cells, peroxisome proliferator‐activated receptor gamma coactivator 1 beta, ZFP36L1

## Abstract

Aplastic anaemia (AA) is a life‐threatening hematopoietic disorder characterized by hypoplasia and pancytopenia with increasing fat cells in the bone marrow (BM). The BM‐derived mesenchymal stem cells (MSCs) from AA are more susceptible to be induced into adipogenic differentiation compared with that from control, which may be causatively associated with the fatty BM and defective hematopoiesis of AA. Here in this study, we first demonstrated that levamisole displayed a significant suppressive effect on the in vitro adipogenic differentiation of AA BM‐MSCs. Mechanistic investigation revealed that levamisole could increase the expression of ZFP36L1 which was subsequently demonstrated to function as a negative regulator of adipogenic differentiation of AA BM‐MSCs through lentivirus‐mediated ZFP36L1 knock‐down and overexpression assay. Peroxisome proliferator‐activated receptor gamma coactivator 1 beta (PPARGC1B) whose 3′‐untranslated region bears adenine‐uridine‐rich elements was verified as a direct downstream target of ZFP36L1, and knock‐down of PPARGC1B impaired the adipogenesis of AA BM‐MSCs. Collectively, our work demonstrated that ZFP36L1‐mediated post‐transcriptional control of PPARGC1B expression underlies the suppressive effect of levamisole on the adipogenic differentiation of AA BM‐MSCs, which not only provides novel therapeutic targets for alleviating the BM fatty phenomenon of AA patients, but also lays the theoretical and experimental foundation for the clinical application of levamisole in AA therapy.

## 1 INTRODUCTION

Aplastic anaemia (AA) is a rare and life‐threatening hematopoietic disorder characterized by hypoplasia and pancytopenia with fatty bone marrow (BM).^1^ The pathogenic factors causally associated with AA may include immune abnormality, quantitative and qualitative defects in hematopoietic stem/progenitors cells and altered marrow microenvironment.^2^, ^3^ Bone marrow mesenchymal stem cells (MSCs) can differentiate into osteoblast, adipocytes and chondrocytes, which together constitute the major cellular components of BM microenvironment providing critical support for hematopoiesis.^4^ There is increasing evidences that BM‐MSCs derived from AA patients display decreased proliferation, aberrant morphology, altered transcriptome profile and impaired differentiation.^5^, ^6^, ^7^ Aplastic anaemia‐derived bone marrow mesenchymal stem cells are more susceptible to be induced to differentiate into adipocyte at the expense of osteoblast,^8^ which, to some degree, may explain the fatty phenomenon in AA BM and defects in hematopoietic support. Despite considerable progress, the role of BM‐MSCs‐mediated microenvironment changes in AA pathogenesis and the molecular mechanism of AA MSCs prone to adipogenic lineage remain to be determined.

Standard front line treatment for AA is either immunosuppressive therapy (IST) or hematopoietic stem cell transplantation (HSCT), which is often accompanied by incomplete recovery, relapse and transplant‐related complications, such as chronic or acute graft‐vs‐host‐diseases (GVHD).^9^, ^10^ Developing new therapeutic strategy for AA is of great significance. Levamisole was originally designed for antihelminthic applications in 1971 and has subsequently been reported to have a broad range of immunomodulatory effects.^11^, ^12^ Li et al^13^ first conducted a prospective trial to treat moderate AA patients by a novel immunosuppressive strategy of cyclosporine alternatively combined with levamisole and achieved encouraging results. However, the molecular mechanism is not clear.

Here in this study, we used levamisole to treat AA BM‐MSCs in vitro and found that levamisole could significantly suppress adipogenic differentiation of AA BM‐MSCs. Mechanistic investigation reveals that ZFP36L1 functions downstream of levamisole and mediates the suppressive effect of levamisole on adipogenesis of AA BM‐MSCs through post‐transcriptional control of peroxisome proliferator‐activated receptor gamma coactivator 1 beta (PPARGC1B) expression. Our findings may provide a promising scheme and potential targets for AA therapy.

## 2 MATERIALS AND METHODS

### 2.1 Cell culture

Mesenchymal stem cells were isolated from the BM of AA patients and healthy controls and cultured in Dulbecco's modified Eagle's medium (DMEM) supplemented with 10% foetal bovine serum (FBS; Gibco). Seven AA patients (4 males and 3 females aged 16‐68) and 6 controls (3 male and 3 female aged 27‐56) were enrolled in this study. The AA patients were diagnosed according to the guidelines previously reported.^14^ All the human samples were obtained from haematology department of the affiliated hospital of Jining Medical University and informed consent to perform the biological studies was obtained from the examined subjects and the related study was approved by the Ethics Committees of the hospitals and the Institutional Review Board of Jining Medical University. 293TN cells were cultured in DMEM with 10% FBS.

### 2.2 Adipogenic induction and levamisole treatment

Adipogenic differentiation was performed according to the method described elsewhere with minor modifications.^15^ The MSCs were seeded into 12‐well plates. Upon reaching confluence, the MSCs were changed into adipogenic medium (AM) composed of DMEM with 10% FBS, 1 μmol/L dexamethasone, 0.01 mg/mL insulin, 100 μg/mL indomethacin and 0.5 mmol/L 3‐isobutyl‐1‐methyl‐xanthine for 14 days (all purchased from Sigma, St. Louis, MO, USA). Levamisole (Sigma) treatment was performed by adding levamisole into the AM at final concentration of 150 μg/mL.

### 2.3 Oil red O staining

The cells were rinsed with PBS twice in the plates after discarding the supernatant and fixed with 4% paraformaldehyde at room temperature for 20 minutes. The cells were then washed with PBS and stained with oil red O (Solarbio) for 20 minutes followed by washing with PBS. Lipid droplets were observed and photographed using an IX71 Olympus microscope (Olympus, Tokyo, Japan). Quantification of the staining was performed using Image‐Pro Plus 6.0 software.

### 2.4 RNA extraction and qRT‐PCR analysis

Total RNA was extracted from cell samples using TRIzol Reagent (Invitrogen) and quantified using the NanoDrop 2000 spectrophotometer (Thermo Scientific, Bremen, Germany). The first strand of cDNA was synthesized using M‐MLV reverse transcriptase (Invitrogen) according to the manufacturer's instructions. Oligo (dT) was used as the primer for reverse transcription of mRNAs. qRT‐PCR was performed in a Bio‐Rad CFX‐96 System (Bio‐Rad, Foster City, CA, USA) using the SYBR Premix (CWBio). The primers used for reverse transcription and qRT‐PCR were listed in Table S1.

### 2.5 Plasmid construction

The ZFP36L1 cDNA was amplified and cloned into pmiRNA1 (System Biosciences, SBI, CA, USA) and pcDNA6 (Invitrogen) to get its expression plasmids. The fragment of PPARGC1B 3′ untranlated regions (UTR) was inserted into modified pcDNA6‐GFP plasmid (Invitrogen). The shRNA sequences for ZFP36L1 and nontarget control were synthesized, annealed and inserted into pll3.7 (Addgene). The shRNA sequences for PPARGC1B were synthesized, annealed and inserted into pSIH‐H1 (System Biosciences, SBI). The primers and oligonucleotides used for plasmid construction were listed in Table S2.

### 2.6 Lentivirus production and cell infection

The recombination lentiviruses for over‐expression and knockdown were produced using the pmiRNA1‐ and pll3.7/pSIH‐H1‐based constructs. Lentivirus packaging was performed using the pPACKH1™ Lentiviral Vector Packaging Kit (LV500A‐1; System Biosciences, SBI) according to the manufacturer's instructions. The culture medium supernatant containing the virus particles was directly used to infect the BM‐MSCs in 12‐well plates with 5 μg/mL polybrene (Sigma Aldrich). After 24‐hour infection, the cells were replaced with fresh complete medium and induced towards adipogenic differentiation.

### 2.7 GFP reporter assay

Two hundred and ninety‐three TN cells were cotransfected with pcDNA6‐GFP‐PPARGC1B (or pcDNA6‐GFP) and pcDNA6‐ZFP36L1 (or pcDNA6) using UltraFection 2.0 (Beijing 4A Biotech) in 6‐well plates. The transfection medium was replaced with complete medium after 5‐6 hours. The cells were cultured at 37°C in 5% CO_2_ for an additional 24‐48 hours. Then, the GFP expression pictures were observed and captured under Fluorescence microscope Olympus IX71 (Olympus). The transfected cells were also collected, rinsed twice with PBS, resuspended in 20‐μL PBS and analysed immediately using a FACSCalibur flow cytometer (BD Biosciences, USA).

### 2.8 Statistical analysis

Student's *t* test (2‐tailed) was performed to analyse the data. Statistical significance was set at *P *<* *.05, as indicated by an asterisk (*P <* *.05; **P <* *.01).

## 3 RESULTS

### 3.1 AA BM‐MSCs exhibits increased adipogenic capacity relative to control BM‐MSCs

**Figure 1:**
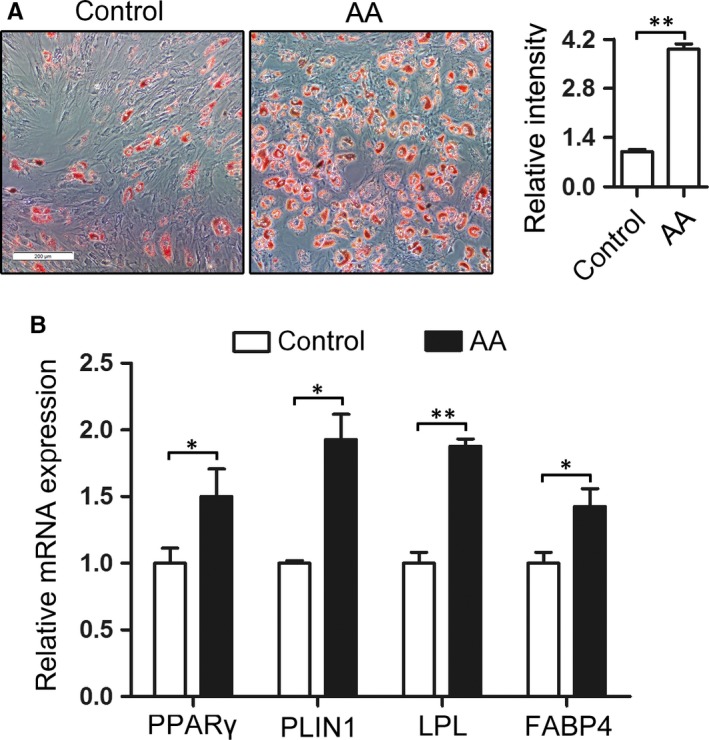
BM‐MSCs derived from AA patients have increased adipogenic capacity relative to that from health controls. The 3rd passage BM‐MSCs derived from AA patients and controls were incubated with adipogenic medium for 14 d. A, The cells were stained with oil red O and presented in the left, and the relative quantification of the staining was analysed and shown in the right. B, Adipogenic markers including PPARγ, PLIN1, LPL and FABP4 were detected using qRT‐PCR. Actin was used as a loading control. *P <* *.05 and **P <* *.01, Student's *t* test. AA, aplastic anaemia; BM, bone marrow; MSCs, mesenchymal stem cells

Aplastic anaemia patients are often characterized by increasing fat cells in the BM. To test whether this is associated with the differentiation alteration of BM‐MSCs towards adipogenic lineage, we first compared the adipogenic capacity of BM‐MSCs derived from AA patients and healthy controls. The MSCs from the 2 groups (control and AA) were cultured in vitro with AM for 14 days, and then the cells were stained with oil red O and also collected for qRT‐PCR analysis to detect the mRNA expression of adipogenic differentiation markers. As shown in Figure 1, the BM‐MSCs in AA group exhibited more lipid droplets (Figure 1A) and higher expression of adipogenic differentiation markers (PPARγ, PLIN1, LPL and FABP4; Figure 1B) compared with that in control group, indicating increased adipogenic capacity of AA BM‐MSCs relative to control. The result obtained is also consistent with the previous literatures,^8^ and to some extent, explained the fatty phenomenon of AA marrow.

### 3.2 Levamisole inhibits the adipogenic differentiation of AA BM‐MSCs

**Figure 2:**
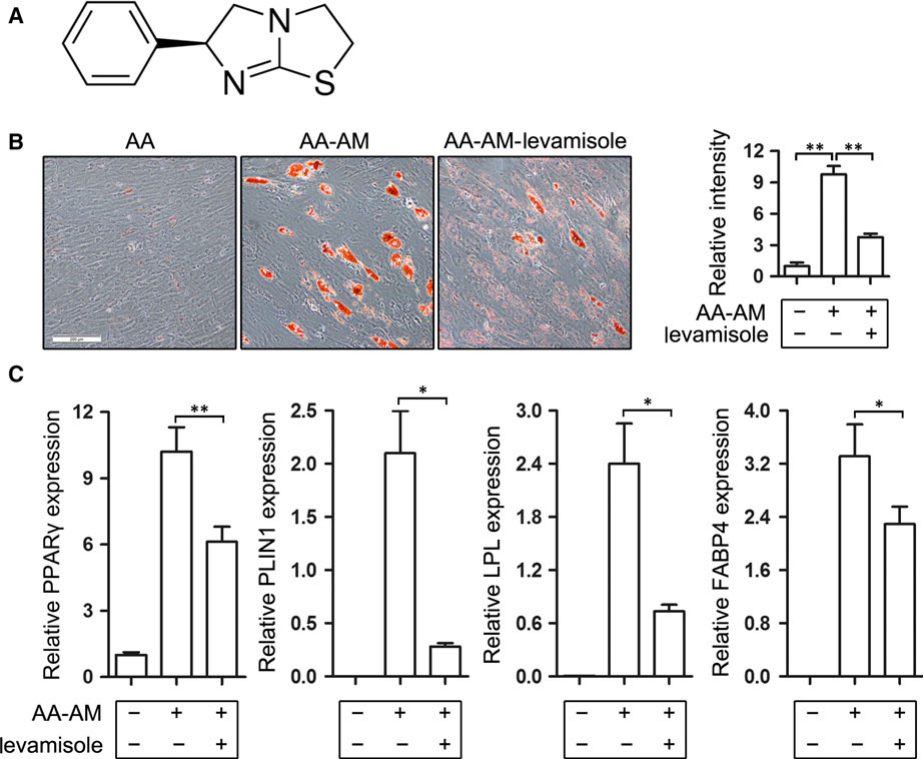
Levamisole inhibits adipogenic differentiation of AA‐derived BM‐MSCs. A, Chemical structure of levamisole. B, AA BM‐MSCs were separated into 3 groups and cultured with and without adipogenic medium (AM) or levamisole (AA, AA‐AM, AA‐AM‐levamisole) for 14 d. The cells were stained with oil red O and presented in the left, and the relative quantification of the staining was analysed and shown in the right. C, Adipogenic markers including PPARγ, PLIN1, LPL and FABP4 were detected using qRT‐PCR. Actin was used as a loading control. *P <* *.05 and **P <* *.01, Student's *t* test. AA, aplastic anaemia; BM, bone marrow; MSCs, mesenchymal stem cells

To seek a promising drug candidate which may be used to inhibit adipogenesis and improve BM microenvironment of AA, we tested a series of small molecules using the in vitro adipogenic differentiation model of AA BM‐MSCs and finally focused on levamisole whose chemical structure was shown in Figure 2A. Aplastic anaemia‐derived bone marrow mesenchymal stem cells were cultured with and without AM for 14 days, and levamisole was concomitantly added into another AM‐cultured AA BM‐MSCs group. Adipogenic induction prompted the formation of lipid droplets (Figure 2B) and the expression of adipogenic differentiation markers (PPARγ, PLIN1, LPL and FABP4) (Figure 2C), which was significantly repressed by the concomitant treatment of levamisole (Figure 2B,C). The results implied that levamisole may be used as a candidate drug for AA therapy through inhibiting adipogenesis of AA BM‐MSCs.

### 3.3 ZFP36L1 shows increased expression upon levamisole treatment and acts as a negative regulator of adipogenic differentiation

**Figure 3:**
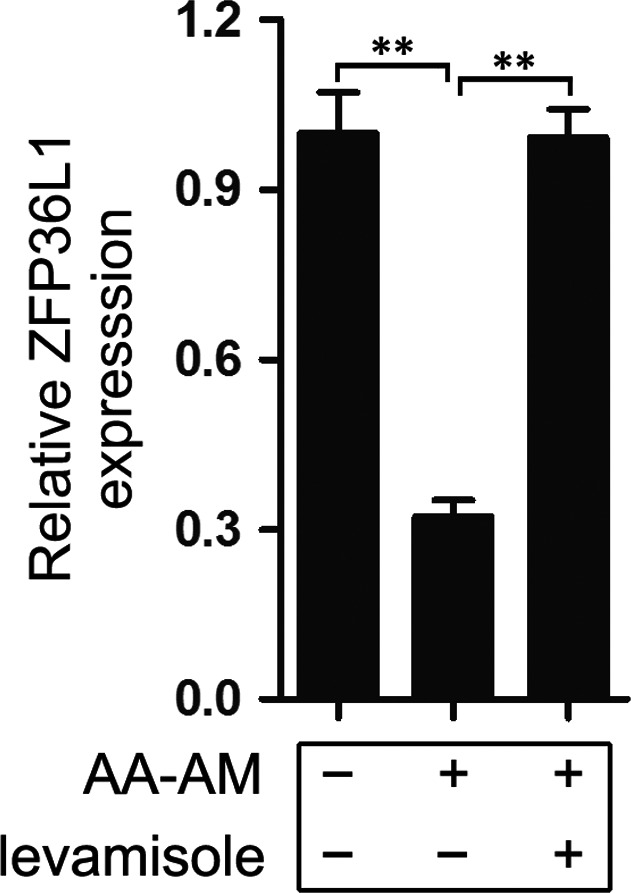
ZFP36L1 is identified as a potential downstream target of levamisole. AA BM‐MSCs were induced into adipogenic lineage with and without levamisole treatment and ZFP36L1 mRNA expression was detected using qRT‐PCR. Actin was used as a loading control. **P <* *.01, Student's *t* test. AA, aplastic anaemia; BM, bone marrow; MSCs, mesenchymal stem cells

**Figure 4:**
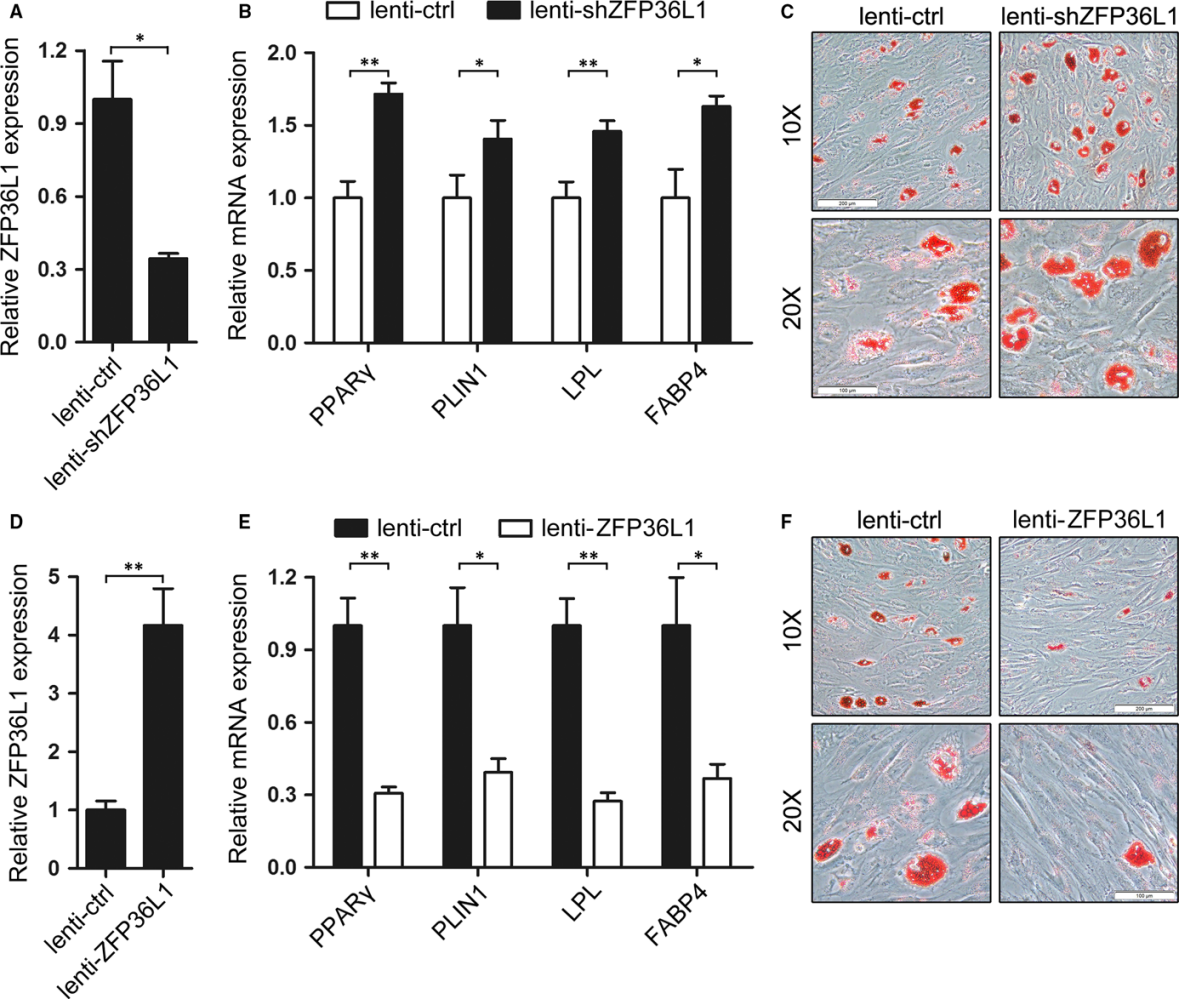
ZFP36L1 acts as a negative regulator of adipogenesis. A‐B, qRT‐PCR analyses of ZFP36L1 expression and adipogenic markers PPARγ, PLIN1, LPL and FABP4. AA BM‐MSCs were infected with lenti‐ctrl and lenti‐shZFP36L1 followed by adipogenic induction for 14 d. C, Oil red O staining of the infected and AM‐induced cells. The cells were observed under 10× and 20× magnifications and a representative experiment was presented. D‐E, qRT‐PCR analyses of the expression of ZFP36L1 and adipogenic markers PPARγ, PLIN1, LPL and FABP4 in AA BM‐MSCs infected with lenti‐ZFP36L1 and lenti‐ctrl, respectively, followed by adipogenic induction for 14 d. F, Oil red O staining of the lenti‐ZFP36L1‐ or lenti‐ctrl‐infected and AM‐induced cells. The cells were observed under 10× and 20× magnifications and a representative experiment was presented. *P <* *.05 and **P <* *.01, Student's *t* test. AA, aplastic anaemia; AM, adipogenic medium; BM, bone marrow; MSCs, mesenchymal stem cells

To reveal the underlying mechanism of levamisole in suppressing adipogenic differentiation of AA BM‐MSCs, we first consulted the gene expression profiles of adipogenic differentiation and AA patients published before^16^, ^17^, ^18^ and focused the differentially expressed genes which may have regulatory roles in adipogenesis. Finally, *ZFP36L1* was identified as a potential target which exhibited decreased expression during the adipogenic induction of AA BM‐MSCs and recovered to original level upon levamisole treatment (Figure 3), indicating that *ZFP36L1* may function as a regulatory molecule in the downstream of levamisole. To further investigate the role of *ZFP36L1* in adipogenesis, we make use of the recombined lentiviruses that express specific short hairpin RNA (shRNA) for *ZFP36L1* (lenti‐shZFP36L1) or *ZFP36L1* (lenti‐ZFP36L1) to infect AA BM‐MSCs followed by adipogenic induction for 14 days. qRT‐PCR analysis revealed that lenti‐shZFP36L1 infection remarkably decreased ZFP36L1 mRNA expression (Figure 4A) which resulted in significant up‐regulation of the mRNA levels of the adipogenic differentiation markers (PPARγ, PLIN1, LPL and FABP4; Figure 4B) as compared with the lenti‐control (lenti‐ctrl) infection. Besides, oil red O staining demonstrated that lenti‐shZFP36L1 infection also exhibited more lipid droplets compared with the lenti‐ctrl infection (Figure 4C). These results demonstrated that knockdown of ZFP36L1 further facilitated the in vitro*‐*induced adipogenic differentiation of AA BM‐MSCs. On the other hand, enforced expression of ZFP36L1 impaired the adipogenic differentiation of AA BM‐MSCs. Compared with the lenti‐ctrl‐infected AA BM‐MSCs, the lenti‐ZFP36L1‐infected cells exhibited a significant over‐expression of ZFP36L1 (Figure 4D) which decreased the mRNA levels of adipogenic differentiation markers (PPARγ, PLIN1, LPL and FABP4; Figure 4E). Besides, the over‐presence of ZFP36L1 resulted in less formation of droplets (Figure 4F). Collectively, these results suggested ZFP36L1's function as an important negative regulator in adipogenic differentiation of AA BM‐MSCs.

### 3.4 PPARGC1B mRNA is identified as a direct target of ZFP36L1

**Figure 5:**
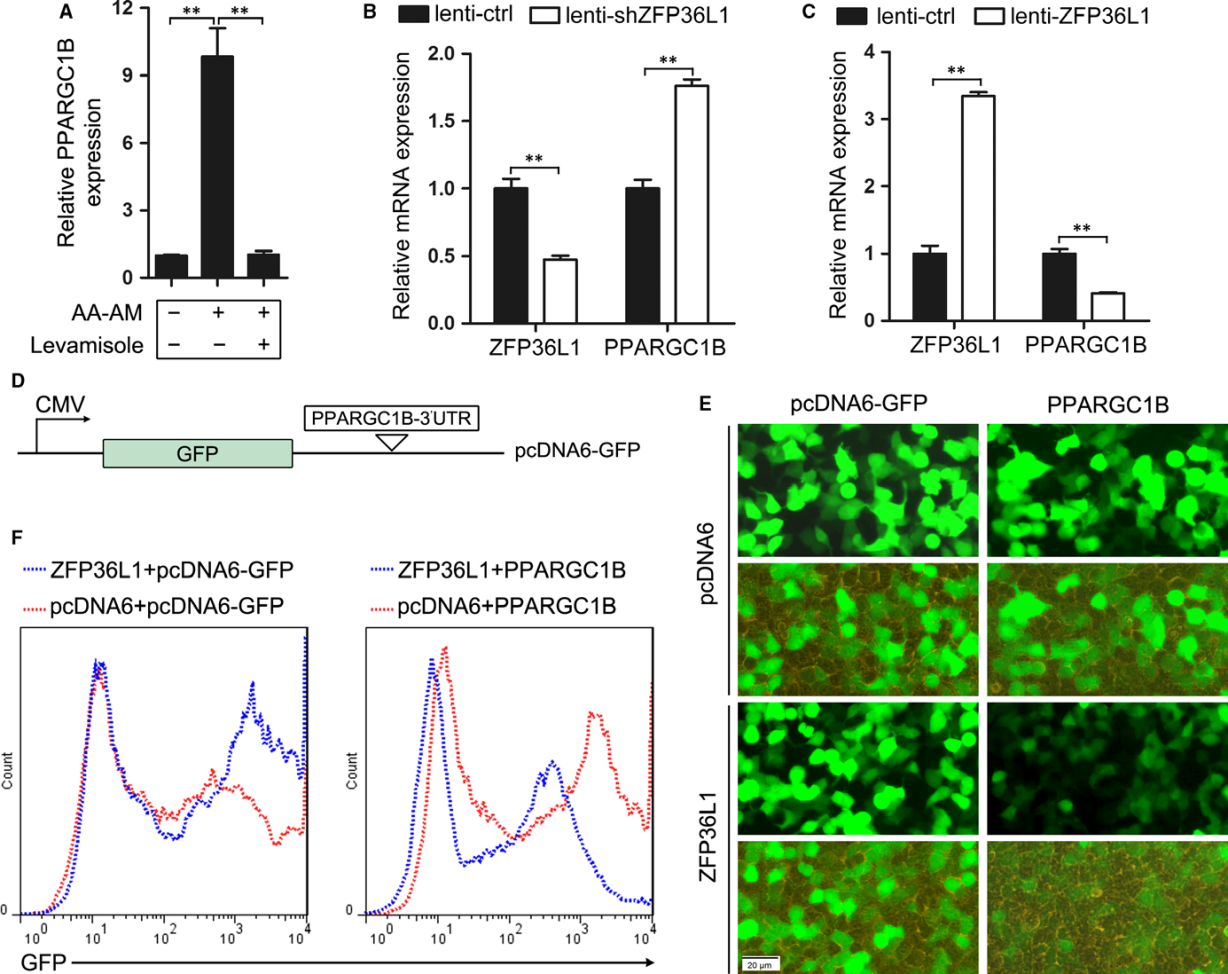
PPARGC1B mRNA is verified as a direct target of ZFP36L1. A, PPARGC1B expression was detected using qRT‐PCR during the adipogenic differentiation with and without levamisole treatment. B‐C, The mRNA level of ZFP36L1 and PPARGC1B was determined in AA BM‐MSCs infected with lenti‐shZFP36L1 (or lenti‐ctrl) and lenti‐ZFP36L1 (or lenti‐ctrl), respectively. D, Schematic outline of PPARGC1B‐GFP reporter construct. E‐F, GFP reporter assay. 293TN cells were co‐transfected with each pcDNA6‐GFP‐based constructs (pcDNA6‐GFP and PPARGC1B) and pcDNA6‐ZFP36L1 (or pcDNA6). The relative GFP expression was presented as fluorescence pictures (E) and also analysed by flow cytometry (F). **P <* *.01, Student's *t* test. AA, aplastic anaemia; BM, bone marrow; MSCs, mesenchymal stem cells; PPARGC1B, peroxisome proliferator‐activated receptor gamma coactivator 1 beta

ZFP36L1 is a RNA binding protein that mainly binds to the adenine‐uridine‐rich elements (AREs) in the 3′‐UTRs and promotes the decay of the target RNAs.^19^ To search for downstream targets of ZFP36L1, we first consulted the AREsite (http://nibiru.tbi.univie.ac.at/AREsite2/welcome)^20^ and downloaded the mRNAs bearing UUAUUUAUU motif in their 3′UTRs, which is implicated by previous work that ZFP36L2, a ZFP36L1 homolog, binds to UUAUUUAUU motif with priority.^21^ There are 1393 mRNAs containing UUAUUUAUU motif. We first focused LPL and PPARGC1B that have been reported to be associated with adipogenesis.^22^, ^23^ LPL and PPARGC1B displayed increased expression during adipogenic differentiation of AA BM‐MSCs, which was significantly repressed by levamisole treatment (Figures 2C and 5A). The expression of LPL and PPARGC1B is opposite to that of ZFP36L1 upon adipogenic induction and levamisole treatment. The influence of ZFP36L1 on LPL and PPARGC1B mRNA levels was also determined in AA BM‐MSCs infected with lenti‐shZFP36L1 (or lenti‐ctrl) and lenti‐ZFP36L1 (or lenti‐ctrl), respectively. The results demonstrated that ZFP36L1 negatively regulates LPL and PPARGC1B mRNA expression (Figures 4B,E and 5B,C). To further confirm whether LPL and PPARGC1B are direct targets of ZFP36L1, the fragments of LPL and PPARGC1B containing ZFP36L1 binding motif were cloned into pcDNA6‐GFP for GFP reporter assay, as depicted in Figure 5D. The GFP expression was presented as fluorescence pictures and flow cytometry analysis (Figure 5E,F), which demonstrated that ZFP36L1 negatively regulates GFP expression in PPARGC1B‐3′UTR‐dependant manner. Similar results were not observed in LPL‐3′UTR‐GFP reporter assay (Figure S1), implying that LPL may not be a direct target of ZFP36L1.

### 3.5 Knockdown of PPARGC1B impairs adipogenic differentiation of AA BM‐MSCs

**Figure 6:**
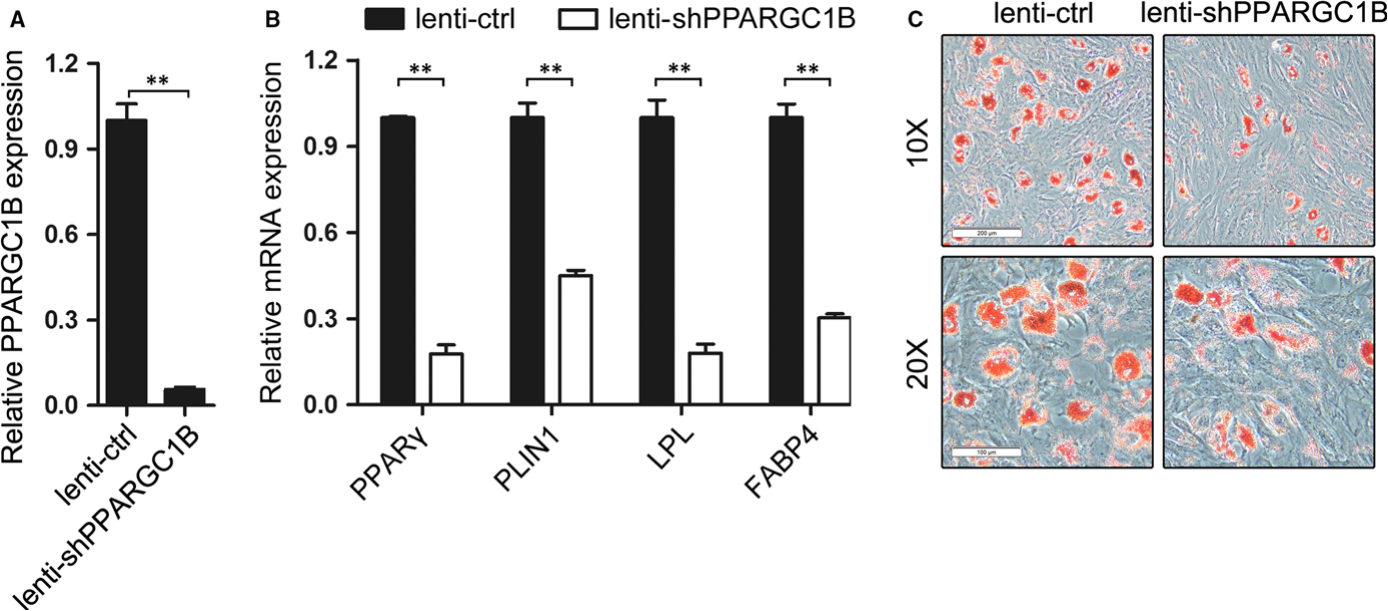
Knockdown of PPARGC1B impairs adipogenic differentiation of AA BM‐MSCs. A‐B, qRT‐PCR analyses of the expression of PPARGC1B and adipogenic markers PPARγ, PLIN1, LPL and FABP4 in AA BM‐MSCs infected with lenti‐shPPARGC1B and lenti‐ctrl, respectively, followed by adipogenic induction for 14 d. C, Oil red O staining of the lenti‐shPPARGC1B‐ or lenti‐ctrl‐infected and AM‐induced cells. The cells were observed under 10× and 20× magnifications and a representative experiment was presented. **P <* *.01, Student's *t* test. AA, aplastic anaemia; AM, adipogenic medium; BM, bone marrow; MSCs, mesenchymal stem cells; PPARGC1B, peroxisome proliferator‐activated receptor gamma coactivator 1 beta

*PPARGC1B* was originally identified by virtue of its function to stimulate the activity of several transcription factors and nuclear receptors.^24^ To investigate the effect of *PPARGC1B* on adipogenic differentiation, we make use of the recombined lentivirus that express specific short hairpin RNA for *PPARGC1B* (lenti‐shPPARGC1B) to infect AA BM‐MSCs followed by adipogenic induction for 14 days. qRT‐PCR analysis revealed that lenti‐shPPARGC1B infection significantly decreased PPARGC1B mRNA expression (Figure 6A), which resulted in significant down‐regulation of the mRNA levels of the adipogenic differentiation markers (PPARγ, PLIN1, LPL and FABP4; Figure 6B) as compared with the lenti‐ctrl infection. Besides, oil red O staining indicated that lenti‐shPPARGC1B infection exhibited less formation of lipid droplets compared with the lenti‐ctrl infection (Figure 6C). These results demonstrated that knock‐down of PPARGC1B impaired adipogenic differentiation of AA BM‐MSCs.

### 3.6 ZFP36L1‐mediated post‐transcriptional control of PPARGC1B expression underlies the suppressive effect of levamisole on adipogenic differentiation of AA BM‐MSCs

**Figure 7:**
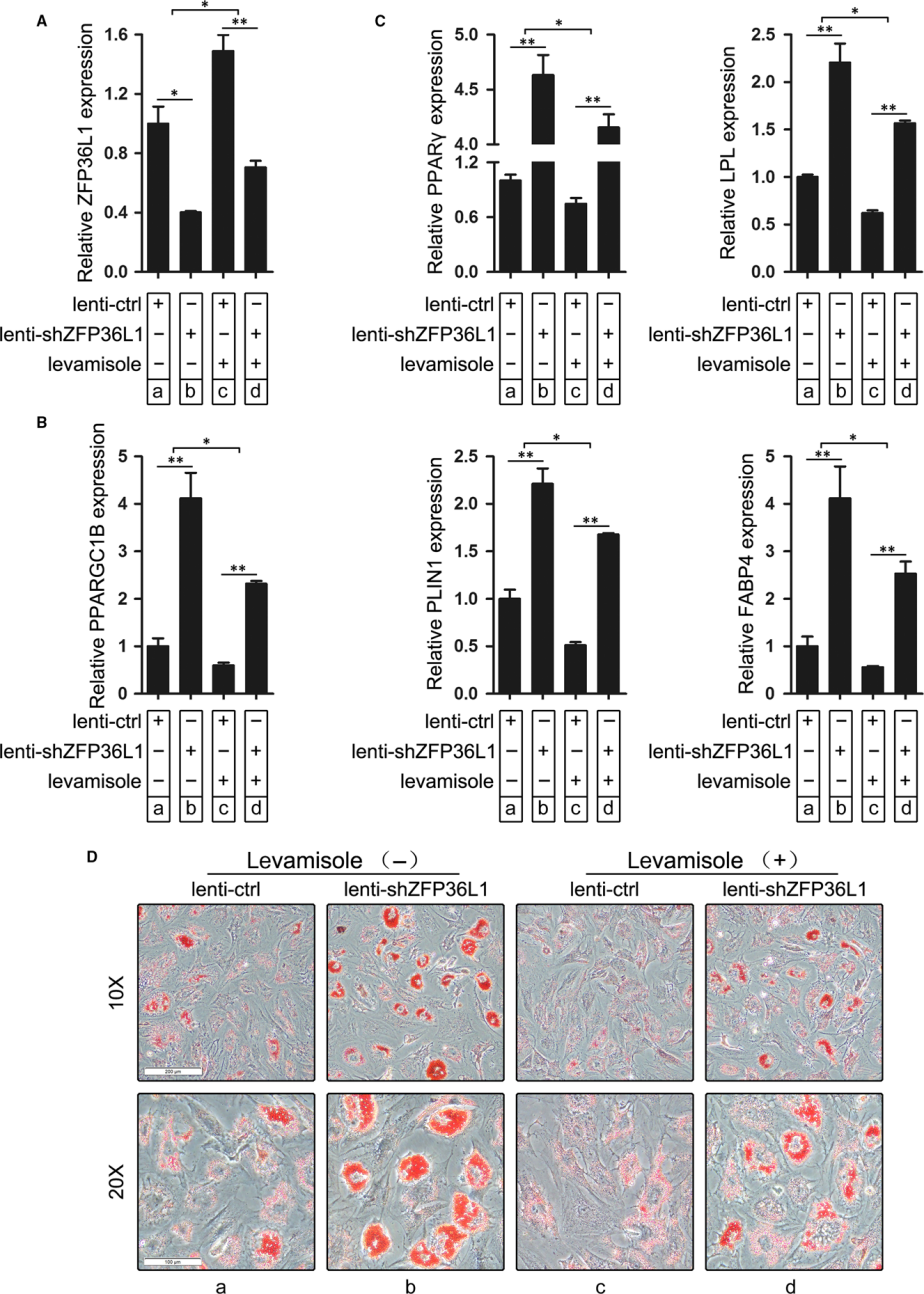
ZFP36L1‐PPARGC1B axis mediates the suppressive effect of levamisole on adipogenic differentiation of AA BM‐MSCs. A‐B, ZFP36L1 and PPARGC1B mRNA expression were evaluated in AA BM‐MSCs infected with lenti‐shZFP36L1 (or lenti‐ctrl) followed by adipogenic induction with levamisole treatment or not. C, Adipogenic markers PPARγ, PLIN1, LPL and FABP4 were detected using qRT‐PCR in infected and AM/levamisole‐treated AA BM‐MSCs. D, Oil red O staining of the lenti‐shZFP36L1‐ or lenti‐ctrl‐infected and AM/levamisole‐treated AA BM‐MSCs. The cells were observed under 10× and 20× magnifications and a representative experiment was presented. *P <* *.05 and **P <* *.01, Student's *t* test. AA, aplastic anaemia; AM, adipogenic medium; BM, bone marrow; MSCs, mesenchymal stem cells; PPARGC1B, peroxisome proliferator‐activated receptor gamma coactivator 1 beta

To further demonstrate that levamisole inhibits adipogenic differentiation of AA BM‐MSCs through *ZFP36L1‐PPARGC1B* axis, we performed rescue assay. Aplastic anaemia‐derived bone marrow mesenchymal stem cells were infected with lenti‐shZFP36L1 or lenti‐ctrl, and 24 hours later, the cells were cultured with adipogenic differentiation medium with and without levamisole treatment for 14 days. As expected, infection with lenti‐shZFP36L1 reversed the up‐regulation of ZFP36L1 caused by levamisole treatment (Figure 7A, d vs c, b vs c). Contrary to ZFP36L1 expression, levamisole‐induced down‐regulation of PPARGC1B was completely compensated by PPARGC1B up‐regulation resulted from lenti‐shZFP36L1 infection (Figure 7B, d vs c, b vs c). Consistent with PPARGC1B expression, lenti‐shZFP36L1‐mediated ZFP36L1 down‐regulation impaired the suppressive effect of levamisole on adipogenic differentiation of AA BM‐MSCs, which is presented as expression of adipogenic differentiation markers (PPARγ, PLIN1, LPL and FABP4; Figure 7C, d vs c, b vs c) and formation of droplets evaluated through oil red O staining (Figure 7D, d vs c, b vs c). These results demonstrated that levamisole may suppress adipogenesis of AA BM‐MSCs through ZFP36L1‐mediated post‐transcriptional control of PPARGC1B expression.

## 4 DISCUSSION

Aplastic anaemia is often considered as an immune‐mediated BM failure syndrome, characterized by defective hematopoiesis and increasing fat cells in the BM.^1^ Accumulating evidences indicated that MSCs‐mediated dysfunction of BM microenvironment plays a significant role in the pathogenesis of AA in view of the immunomodulatory function and hematopoietic support of MSCs and their derivatives.^25^ It is known that the adipogenic and osteogenic differentiation of MSCs is a dynamic process and well balanced in normal BM, the imbalance of which may lead to diseases.^26^, ^27^ Previous studies have suggested that BM adipocytes are predominantly negative regulators of the BM microenvironment and are less supportive to hematopoiesis than those of other cell types derived from MSCs, such as osteoblasts.^28^, ^29^ The BM of AA patients often exhibits increasing adipocytes and decreasing osteoblasts,^3^ which may be causatively associated with the abnormal hematopoietic niche and impaired hematopoiesis. Here in this study, we confirmed that AA BM‐MSCs are more susceptible to be induced into adipogenic differentiation compared with control BM‐MSCs, which, to some extent, accounts for the fatty phenomenon and defective hematopoiesis in AA marrow.

In addition to first‐line standard treatment for AA, such as IST and HSCT,^30^, ^31^ developing small novel molecules which could be used to correct the differentiation imbalance of AA BM‐MSCs or improve the AA BM microenvironment is of great value. Cheng et al^32^ reported that arsenic trioxide (ATO) exerts its clinical efficacy in treating AA patients by inhibiting adipogenic differentiation and facilitating osteogenic differentiation through increasing BMP4 expression, implicating its potential wide‐spread clinical application in AA therapy. In this study, we tested a series of small molecules in the in vitro adipogenic differentiation model of AA BM‐MSCs and found that only levamisole displayed significant suppressive effects on adipogenesis. Levamisole, originally designed for parasitic infections, was reported by Li et al^13^ and Wang et al^33^ for its clinical use in moderate and severe AA therapy. As to whether levamisole could influence the immunomodulatory function and osteogenic differentiation of AA BM‐MSCs, it is still to be determined in our subsequent work.

RNA binding proteins are a kind of post‐transcriptional regulator and have been implicated to influence various aspects of RNA metabolism and participate in many physiological and pathological processes, including hematopoiesis^34^ and adipogenesis.^35^ To reveal the underlying mechanism of levamisole in inhibiting adipogenesis, we unexpectedly found that levamisole could increase the expression of ZFP36L1 which is an AREs binding protein. Adenine‐uridine‐rich elements are a group of loosely defined adenylate‐uridylate‐rich instability determinants with sizes ranging from 50 to 150 nucleotides mainly found in the 3′UTRs of mRNAs.^36^ ZFP36L1 belongs to the zinc finger protein family and has been implicated to bind AREs in the 3′UTRs of mRNAs through its RNA binding domain, resulting in destabilization of the target RNAs.^37^ As a post‐transcriptional regulator, ZFP36L1 has been reported to participate in erythroid differentiation and monocyte/macrophage differentiation by directly targeting stat5b and CDK6 expression,^38^, ^39^ respectively. ZFP36L1 can also act as a tumour suppressor to influence tumorigenesis through negatively regulating VEGFA and Bcl2 expression.^40^, ^41^ However, the role of ZFP36L1 in adipogenesis has only been implicated by the report that it could enhance the degradation of PPARγ2 mRNA transcript in 3T3‐L1 preadipocytes.^42^ In this study, through lentiviruses‐mediated *ZFP36L1* over‐expression and knockdown assay, we demonstrated that *ZFP36L1* functions in the downstream of levamisole and acts as a negative regulator in the adipogenic differentiation of the human AA BM‐MSCs. Further mechanistic analysis revealed that PPARGC1B mRNA who contains AREs in its 3′UTRs is verified as a direct target of ZFP36L1. As a PPARγ coactivator, *PPARGC1B* has previously been reported to be correlated with fatty acid oxidation and mitochondrial biogenesis,^43^ and its expression and positive regulatory role in adipogenesis of AA BM‐MSCs was first uncovered in this work. We also performed rescue assay using levamisole and ZFP36L1 shRNA and demonstrated that *ZFP36L1‐PPARGC1B* axis mediated the suppressive effect of levamisole on adipogenic differentiation of AA BM‐MSC. In summary, our results demonstrated that levamisole could inhibit adipogenesis of BM‐MSCs derived from AA patients through ZFP36L1‐mediated post‐transcriptional regulation of PPARGC1B expression by binding to its 3′UTR, which not only provides novel therapeutic targets for alleviating the BM fatty phenomenon of AA patients, but also lays the theoretical and experimental foundation for the clinical application of levamisole in AA therapy.

## CONFLICT OF INTERESTS

The authors declare that they have no competing interests.

## AUTHOR CONTRIBUTION

L.‐L.L., L.L and H.‐H.L. performed experiments and interpreted data. S.‐S.R, C.‐Y.D, P.‐P.C, C.‐L.W, L.‐N.W and X.‐L.C helped collect the samples and performed partial experiments. M.‐T.C and H.Z designed the study, directed the experiments and wrote the manuscript. All authors read and approved the final manuscript.
