# Supplementary material for: Levamisole suppresses adipogenesis of aplastic anaemia‐derived bone marrow mesenchymal stem cells through ZFP36L1‐PPARGC1B axis
Source: J Cell Mol Med. 2018 Jul 11;22(9):4496–506. doi: 10.1111/jcmm.13761 (PMC6111807; doi:10.1111/jcmm.13761)
Supplement: Supplementary file 1 [file JCMM-22-4496-s001.doc]

Supplementary material

**Levamisole suppresses adipogenesis of aplastic anemia-derived bone marrow mesenchymal stem cells through ZFP36L1-PPARGC1B axis**

Lu-Lu Liu ^1, †^, Lei Liu ^3, †^, Hai-Hui Liu ^2, †^, Sai-Sai Ren ^3^, Cui-Yun Dou ^3^, Pan-Pan Cheng ^3^, Cui-Ling Wang ^3^, Li-Na Wang ^1^, Xiao-Li Chen ^2^, Hao Zhang ^3*^, Ming-Tai Chen ^1*^


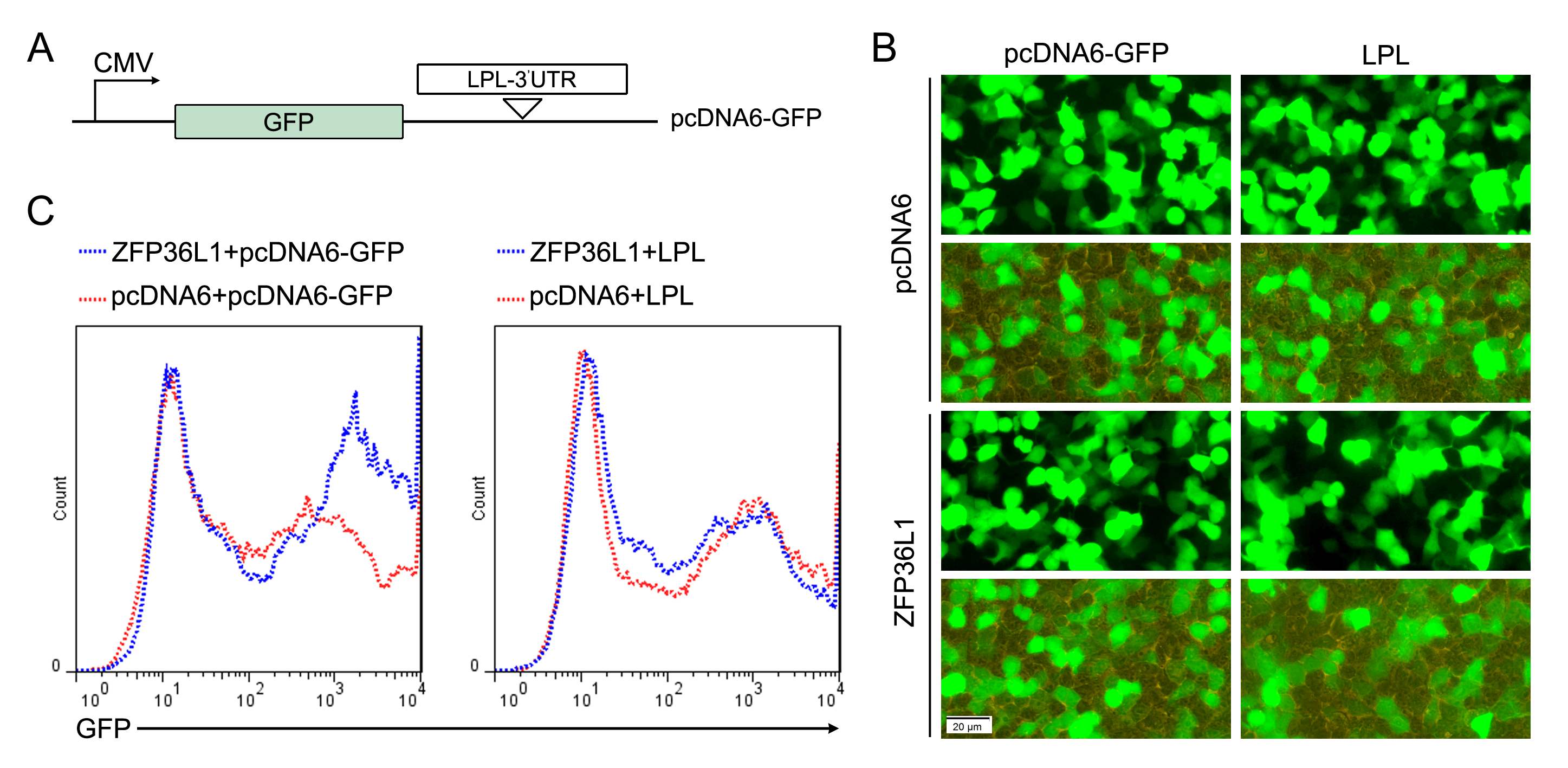


Fig.S1 LPL mRNA is verified not as a direct target of ZFP36L1 through GFP reporter assay. (A) Schematic outline of LPL-GFP reporter construct. (B-C) GFP reporter assay. 293TN cells were co-transfected with each pcDNA6-GFP-based constructs (pcDNA6-GFP and LPL) and pcDNA6-ZFP36L1 (or pcDNA6). The relative GFP expression was presented as fluorescence pictures (B) and also analyzed by flow cytometry (C).

**Table S1.** **Primers used for reverse transcription and qRT-PCR.**

| Name | Primers |
| --- | --- |
| ZFP36L1-F | ATGACCACCACCCTCGTGT |
| ZFP36L1-R | TTTCTGTCCAGCAGGCAACC |
| GAPDH-F | GGAGCGAGATCCCTCCAAAAT |
| GAPDH-R | GGCTGTTGTCATACTTCTCATGG |
| Actin-F | CTGGCACCACACCTTCTACA |
| Actin-R | AGCACAGCCTGGATAGCAAC |
| PPARγ-F | GCTGACCAAAGCAAAGGCG |
| PPARγ-R | GCCCTGAAAGATGCGGATG |
| LPL-F | TCATTCCCGGAGTAGCAGAGT |
| LPL-R | GGCCACAAGTTTTGGCACC |
| PLIN1-F | GCGAGGATGGCAGTCAACAAA |
| PLIN1-R | GCACGCCCTTCTCATAGGCAT |
| FABP4-F | ACTGGGCCAGGAATTTGACG |
| FABP4-R | CTCGTGGAAGTGACGCCTT |
| PPARGC1B-F | GATGCCAGCGACTTTGACTC |
| PPARGC1B-R | ACCCACGTCATCTTCAGGGA |
| oligodT | TTTTTTTTTTTTTTTTTT |

**Table S2.** Primers and oligonucleotides used for plasmid construction.

| Name | Primers and oligonucleotides |
| --- | --- |
| ZFP36L1-CEF1 | GGATCCTCCAGGTGCAGGGGCTT ( EcoR Ⅰ) |
| ZFP36L1-CER1 | GCGGCCGCCCATCCAGGACAGAATG ( Not Ⅰ) |
| PPARGC1B-EF1 | TGAAACCAACACGAGCCCTCT ( EcoR Ⅰ) |
| PPARGC1B-ER1 | CTCGAGTTCCTCCTTGCCTCTTA ( Xho Ⅰ) |
| shcon-F | TGAACTCAAGACCGATATTATTCAAGAGATAATATCGGTCTTGAGTTCTTTTTTC |
| shcon-R | TCGAGAAAAAAGAACTCAAGACCGATATTATCTCTTGAATAATATCGGTCTTGAGTTCA |
| ZFP36L1-shRNA-F | TGTAACAAGATGCTCAACTATTCAAGAGATAGTTGAGCATCTTGTTACTTTTTTC |
| ZFP36L1-shRNA-R | TCGAGAAAAAAGTAACAAGATGCTCAACTATCTCTTGAATAGTTGAGCATCTTGTTACA |
| PPARGC1B-shRNA-F | GATCCGTACAGAACTACATAAGCATTCAAGAGATGCTTATGTAGTTCTGTACTTTTTG |
| PPARGC1B-shRNA-R | AATTCAAAAAGTACAGAACTACATAAGCATCTCTTGAATGCTTATGTAGTTCTGTACG |
